# Bi_2_WO_6_–BiOCl heterostructure with enhanced photocatalytic activity for efficient degradation of oxytetracycline

**DOI:** 10.1038/s41598-020-75003-x

**Published:** 2020-10-27

**Authors:** Mengfan Guo, Zhaobo Zhou, Shengnan Yan, Pengfei Zhou, Feng Miao, Shijun Liang, Jinlan Wang, Xinyi Cui

**Affiliations:** 1 State Key Laboratory of Pollution Control and Resource Reuse, School of the Environment, Nanjing University, Nanjing University, 163 Xianlin Avenue, Nanjing, 210046 China; 2 National Laboratory of Solid State Microstructures, School of Physics, Collaborative Innovation Center of Advanced, Microstructures Nanjing University, Nanjing, 210093 China; 3 Department of Physics, Southeast University, Nanjing, 211189 China

**Keywords:** Environmental sciences, Energy science and technology, Materials science

## Abstract

The application of BiOCl in photocatalysis has been restricted by its low utilization of solar energy and fast recombination of charge carriers. In this study, zero-dimensional (0D) Bi_2_WO_6_ nanoparticles/two-dimensional (2D) layered BiOCl heterojunction composite was successfully constructed by facile hydrothermal and solvothermal methods. The most favorable sunlight photocatalytic activity was achieved for the as-prepared Bi_2_WO_6_–BiOCl composites with a ratio of 1%. The photocatalytic rate and mineralization efficiency of one typical antibiotic (i.e., oxytetracycline) over 1% Bi_2_WO_6_–BiOCl was about 2.7 and 5.3 times as high as that of BiOCl. Both experimental characterizations and density functional theory (DFT) calculations confirmed that the excellent photocatalytic performance mainly arised from the effective charge separation along the Bi_2_WO_6_ and BiOCl heterojunction interface. The effective electron transfer was driven by the internal electric field at the interfacial junction. In addition, 1% Bi_2_WO_6_–BiOCl exhibited excellent stability, and no apparent deactivation was observed after 4 test cycles. Therefore, the 0D/2D Bi_2_WO_6_–BiOCl heterojunction showed a great potential for the photocatalytic degradation of emerging organic pollutants.

## Introduction

As the rapid progress of industrialization, the accumulation of organic contaminant in natural water body is of a great threaten to global environment and human health. Traditional organic contaminant removal technologies, such as physical adsorption, chemical oxidation, and biological degradation, are usually undesirable due to their low efficiency and high energy consumption^1–3^. Semiconductor-based photocatalysis has drawn great attention for their potential in directly utilizes solar energy for environmental pollution decomposition^4^.

Two-dimensional (2D) lamellar structures are promising photocatalysts owing to their high emission quantum yields, large charge carrier mobility, and short bulk diffusion length^5^. Bismuth oxychloride (BiOCl), with a special layer structure consisting of [Bi_2_O_2_]^2+^ layers sandwiched between two slabs of halogen ions, has shown favorable photocatalytic performance and stability^6^. However, there are still some bottlenecks hindering its practical application, such as wide band gap and fast recombination of electron–hole pairs of single BiOCl^7^. Some efforts have been devoted to deal with those drawbacks, such as controlling exposed crystal facets^8^, varying morphology and size^9,10^, noble metal doping^11,12^, and constructing heterostructures^13–15^, etc. Among these methods, the fabrication of heterostructure is one of the most effective ways. Due to the difference of band gap and position between the two semiconductors, it can be expected that the formation of heterojunction can promote the separation of photogenerated electron–hole pairs and thus improve the photocatalytic efficiency^16^.

As one of the simplest Aurivillius phases, bismuth tungstate (Bi_2_WO_6_) has received a lot of attention due to its special chemistry structure and excellent visible-light response properties^17–19^. Bi_2_WO_6_ has suitable band edges (E_CB_ = 0.03 eV, E_VB_ = 2.93 eV)^20^, and can match well with BiOCl (E_CB_ = − 0.80 eV, E_VB_ = 1.09 eV) to form a heterostructure^13^. Additionally, both Bi_2_WO_6_ and BiOCl belong to the layered Aurivillius family, consisting of [Bi_2_O_2_]^2+^ layers sandwiched between two slabs of [WO_4_]^2−^ or Cl ions, which render them ready to match well with each other^21,22^.

Recently, construction of closely coupled 0D–2D heterojunction is effective to form composite materials with excellent photocatalytic efficiency^23,24^. Firstly, 0D nanoparticles have advantages of large surface area, short charge-migration distance and size-tunable optoelectronics, suggesting their promising photocatalysis potential. Moreover, tight interactions between 0 and 2D components can make 0D nanoparticles more dispersive and stable, while the enhanced charge transfer facilitated by 2D nanosheets can effectively inhibit the recombination of photo-excited charges^25–27^. Therefore, it might be a robust approach to boost the photocatalytic performance by constructing 0D Bi_2_WO_6_ nanoparticle/2D BiOCl nanosheet heterojunction to improve the photocatalytic efficiency.

In the current work, a 0D Bi_2_WO_6_ nanoparticles/2D BiOCl nanosheets heterojunction was fabricated via a facile hydrothermal and solvothermal process. Their photocatalytic performance towards oxytetracycline (OTC) degradation under simulated sunlight irradiation was systematically evaluated, and the photocatalytic degradation of model contaminant (i.e., phenol) was also conducted for comparison. Mechanism of the enhanced photocatalytic activity of Bi_2_WO_6_–BiOCl heterojunction was comprehensively explained by instrumental characterizations (such as photo-luminescence spectra, valence band X-ray photoelectron spectroscopy, and UV–Vis diffuse reflectance spectra) and density functional theoretical (DFT) calculations.

## Experimental section

### *Synthesis of Bi*_*2*_*WO*_*6*_*–BiOCl heterojunction*

Bi_2_WO_6_ nanoparticles were prepared by a modified solvothermal method^28^. Briefly, 1 mmol Bi(NO_3_)_3_·5H_2_O and 0.5 mmol Na_2_WO_4_·2H_2_O were dispersed in 10 mL ethylene glycol (EG), respectively. These two solutions were ultra-sonicated for 30 min to form uniform tungsten source and bismuth source. The tungsten source was then slowly added to bismuth source with continuous stirring. The mixture was transferred into 50 mL Teflon-lined stainless steel autoclave, and heated at 160 °C for 10 h. After being cooled to room temperature, the precipitate was washed with ethanol for three times and dried at 60 °C for 12 h in air.

The Bi_2_WO_6_–BiOCl composites were fabricated by a previously reported method^3^. Briefly, 1 mmol Bi(NO_3_)_3_·5H_2_O and aliquots (i.e. 0.005 mmol, 0.01 mmol, 0.02 mmol, 0.04 mmol) of Bi_2_WO_6_ were added in 10 mL distilled water at room temperature with vigorous stirring and ultra-sonication for 1 h. After that, 10 mL of 0.1 mol L^−1^ KCl aqueous solution was added to Bi(NO_3_)_3_·5H_2_O solution by dropwise. The solution was vigorously stirred for 30 min, and 1 M NaOH was then added to adjust pH value to 6.0. The mixture was stirred for 1 h, poured into a 50 mL Teflon-lined stainless steel autoclave, and heated up to 160 °C for 24 h. After being cooled to room temperature, the white precipitate was collected and washed with distilled water for three times. The washed precipitate was then dried at 60 °C for 12 h in air to get the Bi_2_WO_6_–BiOCl composites. According to Bi_2_WO_6_ mass in the reaction system, Bi_2_WO_6_–BiOCl samples were denoted as 0.5%, 1%, 2%, and 4% Bi_2_WO_6_–BiOCl. The pure BiOCl were also obtained without the addtion of Bi_2_WO_6_ under the same condition. The 1% mixture were prepared by simply mechanical blending of Bi_2_WO_6_ and BiOCl at a ratio of 1%.

### Characterization

The powder X-ray diffraction (XRD) patterns were collected by an X-ray diffractometer (Bruker D-8 Advance) with Cu Ka radiation (λ = 0.15406 nm). The 2θ ranged from 10° to 80° with a scanning rate of 8° min^−1^. The morphologies and compositions of Bi_2_WO_6_, BiOCl, and Bi_2_WO_6_–BiOCl composites were performed by scanning electron microscopy (SEM) and energy dispersive X-ray spectroscopy (EDS) on a FEI Nova-450 scanning electron microscopy. Transmission electron microscopy (TEM), high-resolution transmission electron microscopy (HR-TEM) images, and selected-area electron diffraction (SAED) pattern were recorded in a JEM-200CX instrument with an accelerating voltage 200 kV. X-ray photoelectron spectroscopy (XPS) measurements were carried out by a PHI 5000 Versa Probe spectrometer with an Mg Kα ray source, and the binding energies were calibrated to the C1s peak at 284.6 eV. The specific surface areas of samples were examined by nitrogen adsorption and desorption apparatus (NOVA, Quantachrome, USA) with Brunauer–Emmett–Teller (BET) method. The light absorption properties of the samples were recorded by UV–Vis diffuse reflectance spectra (UV–Vis-DRS, Hitachi UV-3600) with BaSO_4_ as the reference. The photoluminescence spectra (PL) were obtained using Fluorescence spectrometry (HORIBA fluoromax-4) with excitation at 315 nm. The electron spin resonance (ESR) signals of radicals (^·^OH and ^·^O_2_^−^) were tested on the X-band Bruker A-200 spectrometer (Germany).

### Photocatalytic activity measurements

The photocatalytic activities for degradation of oxytetracycline (OTC) and phenol were tested by using a 500 W Xe lamp (CEL-HXF500, AULTT, China) at ambient temperature, and the optical power density was maintained about 42 mW cm^−2^ measured by a radiometer (CEL-NP2000, AULTT). In a typical process, 30 mg of the photocatalyst was dispersed in 30 mL OTC or phenol aqueous solution with a concentration of 20 ppm. Before irradiation, the solution was continuously stirred in the dark for 2 h to ensure the establishment of adsorption–desorption equilibrium. During the degradation test, 1 mL of suspension were taken at given time intervals, centrifuged, and filtered to obtain the suspernatant for OTC or phenol analysis. The concentrations of OTC and phenol were determined by high performance liquid chromatography (HPLC; Waters, e2695, Ireland) with a XBridge™ C18 column (5 μm, 4.6 × 250 mm) and a UV detector operated at 355 nm and 270 nm, respectively. The mobile phase consisted of methanol and water (volume ratio: 60/40) at a flow rate of 1 mL min^−1^ for phenol analysis^29^, and 0.005 mol L^−1^ oxalic acid solution/methanol/acetonitrile (60/20/20, v/v/v) with a flow rate of 0.4 mL min^−1^ for OTC analysis^30^. The mineralization of OTC and phenol were determined by the change of total organic carbon (TOC) in supernant on a total organic carbon analyser (Vario TOC, Elementar).

### Computational method

All the calculations were performed by means of density functional theory (DFT), as implemented in the Vienna Ab-initio Simulation Package (VASP)^31,32^ within the framework of the projector augmented wave (PAW) method^33^. The generalized gradient approximation (GGA) with the Perdew–Burke–Ernzerhof (PBE) functional was utilized to describe the exchange correlation interactions^34^. A 500 eV cutoff for the plane wave basis set was adopted in all computations. Structural relaxations were carried out until the residual forces on atoms less than 0.01 eV Å^−1^. The convergence criterion of self-consistent calculations for ionic relaxations was set to 1 × 10^–5^ eV atom^−1^. The corresponding lattice parameter of unit cell for Bi_2_WO_6_ was calculated as a = 5.55 Å, b = 16.85 Å and c = 5.58 Å and that for BiOCl was a = b = 3.91 Å and c = 7.84 Å, which were in good agreement with previous reports^35,36^. For the Bi_2_WO_6_ (020)/BiOCl (010) interface model, a 3 × 4 Bi_2_WO_6_ (020) surface slab was used to match a 7 × 3 BiOCl (010) surface slab. The lattice mismatch between 3 × 4 Bi_2_WO_6_ (020) and 7 × 3 BiOCl (010) surface slabs is 3.82%.

The vacuum space in the z-direction was set as large as 15 Å to avoid interactions between the repeated slabs. A Monkhorst–Pack special k-point mesh of 1 × 3 × 1 was proposed to carry out geometry optimization and electronic structure calculation. The charge-density difference (Δ*ρ*) was calculated by

$$\Delta \rho = \Delta \rho Bi_{2} WO_{6} \left( {020} \right)/BiOCl \, \left( {010} \right) - \, \Delta \rho BiOCl \, \left( {010} \right) - \, \Delta \rho Bi_{2} WO_{6} \left( {020} \right)$$

where Δ*ρBi*_*2*_*WO*_*6*_* (020)/BiOCl (010)*, Δ*ρBiOCl (010)* and Δ*ρBi*_*2*_*WO*_*6*_* (020)* were the total charge density of the Bi_2_WO_6_ (020)/BiOCl (010) heterostructures, BiOCl (010), and Bi_2_WO_6_ (020) surface slabs, respectively.

## Results and discussion

### Characterization of the catalysts

**Figure 1:**
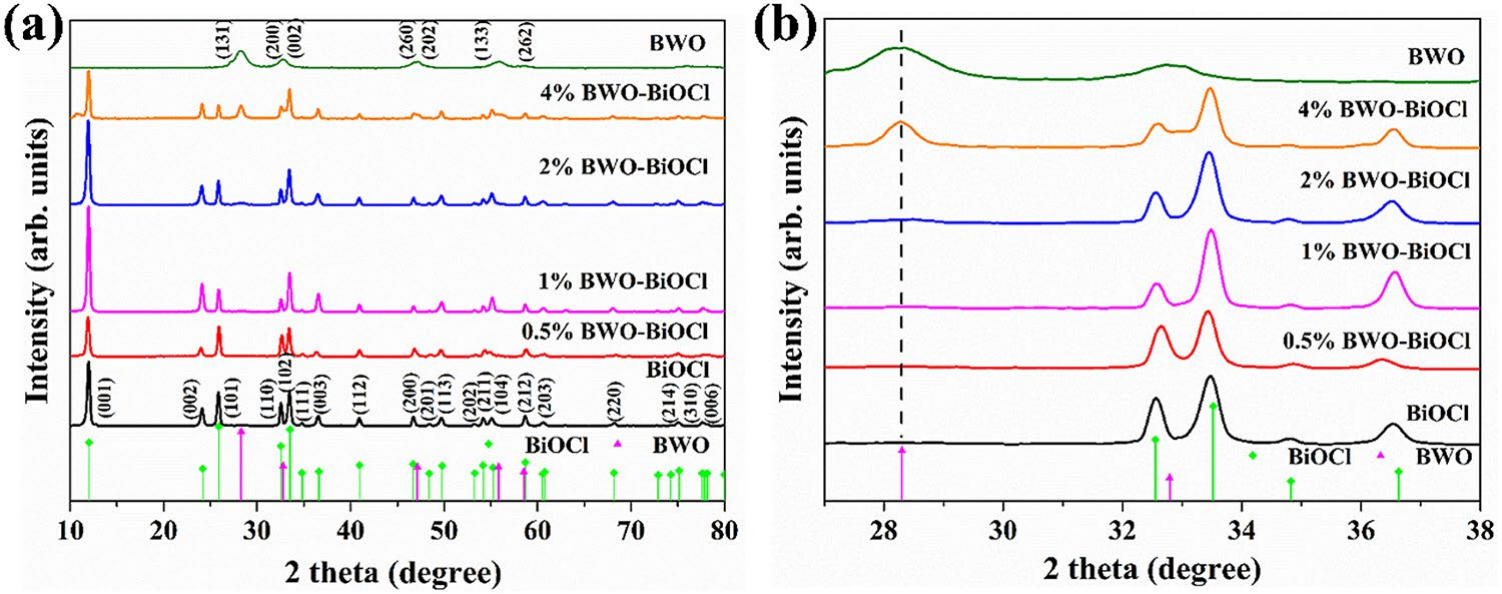
(**a**) XRD and (**b**) enlarged XRD patterns of BiOCl, Bi_2_WO_6_, 0.5%, 1%, 2%, and 4% Bi_2_WO_6_–BiOCl.

The phase structure and purity of the as-prepared Bi_2_WO_6_, BiOCl, 0.5%, 1%, 2% and 4% Bi_2_WO_6_–BiOCl were characterized by power X-ray diffraction (XRD). As shown in Fig. 1a, the peak of all the photocatalysts were sharp and narrow, indicating that the samples possess single-phase and well-identified crystalline structures (Bi_2_WO_6_: JCPDS card no.39–0256; BiOCl: JCPDS card no. 85-0861). For pure Bi_2_WO_6_, a sharp peak at 28.3° was assigned to (131) plane. While a series of typical peaks located in 12.0°, 24.1°, 25.8°, 32.5° and 33.5° were indexed as (001), (002), (101), (110) and (102) of BiOCl. The magnified part of XRD data from 27° to 39° are shown in Fig. 1b. With the augment of Bi_2_WO_6_ proportions, the peak intensity of the Bi_2_WO_6_ (28.3°) gradually increased, and the relative peak intensity of the BiOCl was almost unchange. This indicated that compositing of Bi_2_WO_6_ cannot influence the crystal structure of BiOCl.

**Figure 2:**
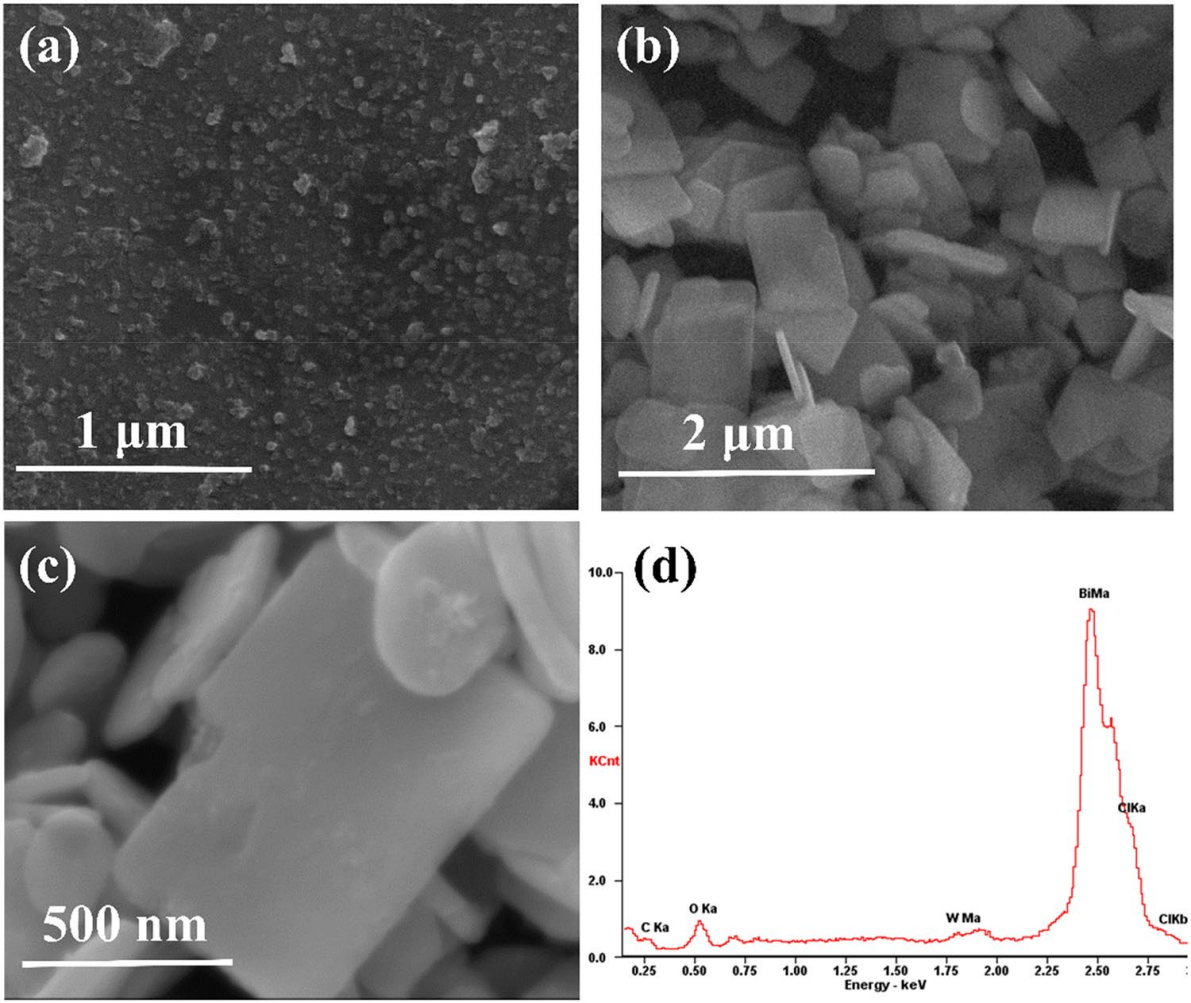
SEM images of (**a**) Bi_2_WO_6_, (**b**) BiOCl nanosheet, (**c**) 1% Bi_2_WO_6_–BiOCl, and (**d**) EDS spetra of 1% Bi_2_WO_6_–BiOCl.

The structure and morphology of the samples were characterized with scanning electron microscopy and energy dispersive X-ray spectroscopy (SEM–EDS) (Fig. 2).

SEM revealed that pure Bi_2_WO_6_ were nanoparticales with size of 5–10 nm, and BiOCl consisted of nanosheets with size of 0.5–1 μm (Fig. 2a,b). On the other hands, the morphology of 1% Bi_2_WO_6_–BiOCl heterostructure is shown in Fig. 2c. The surface element dispersion states of 1% Bi_2_WO_6_–BiOCl was measured by EDS technology. Strong signals from Bi, Cl, O and W elements can be observed in EDS spectra (Fig. 2d). The atomic ratio of Bi:O:Cl:W was 45:36:29:1 in the 1% Bi_2_WO_6_–BiOCl, indicating that Bi_2_WO_6_ was successfully incorporated with BiOCl, which was consistent with XRD data.

**Figure 3:**
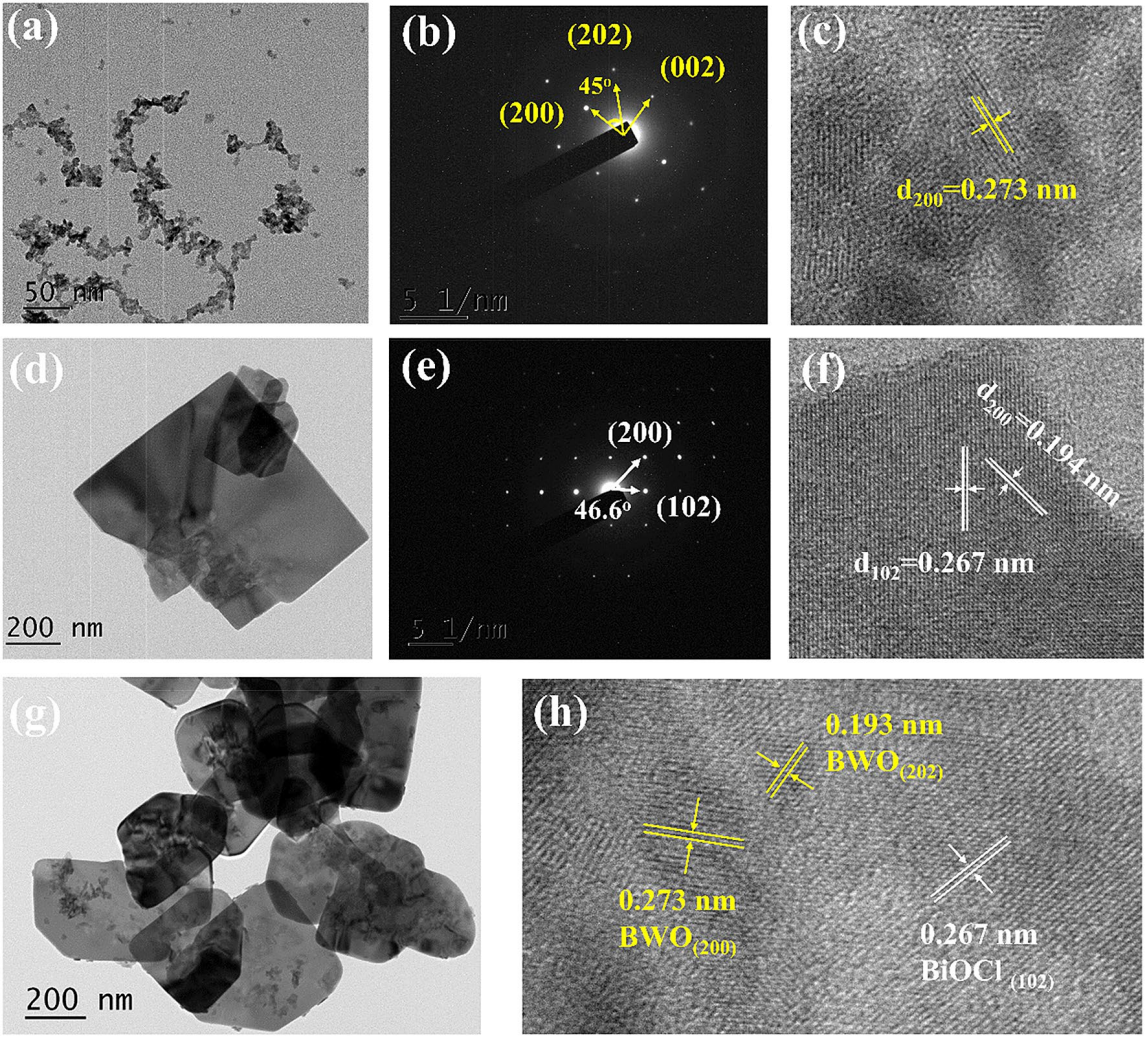
TEM and HR-TEM images of (**a**,**c**) Bi_2_WO_6_, (**d**,**f**) BiOCl nanosheet, and (**g**,**h**) 1% Bi_2_WO_6_–BiOCl composites, SAED patterns of (**b**) Bi_2_WO_6_ and (**e**) BiOCl.

Transmission electron microscopy (TEM) and selected-area electron diffraction (SAED) analyses were applied to further investigate the phase structure of Bi_2_WO_6_ nanoparticles, BiOCl nanosheets, and 1% Bi_2_WO_6_–BiOCl heterojunctions. As shown in Fig. 3a, the Bi_2_WO_6_ sample exhibited nanoparticles with a size of 5–10 nm. The SAED pattern indicated the single-crystalline characteristic of the Bi_2_WO_6_ sample (Fig. 3b). The angle labled in the SAED pattern was 45°, which was in agreement with the theoretical value of the angle between the (200) and (202) planes. The set of diffraction spots can be indexed as the [010] zone axis of orthorhombic Bi_2_WO_6_. Figure 3c is the high-resolution TEM (HR-TEM) of Bi_2_WO_6_ sample, the interplanar lattice spacing of 0.273 nm corresponded to the (200) planes of Bi_2_WO_6_. Thus, it can be found that the main exposed facet of Bi_2_WO_6_ was {020} facets. The TEM image of BiOCl sheets is illustrated in Fig. 3d, and the width of BiOCl was estimated to be 0.5–1 μm. The corresponding SAED pattern (Fig. 3e), indexed as the [010] zone, showed (200) and (102) planes with an interfacial angle of 46.6°, which was identical to the theoretical value. In HR-TEM image (Fig. 3f) of BiOCl nanosheet, the distances between adjacent lattice fringes were measured as 0.194 and 0.267 nm, respectively. This value corresponded to the interplanar distances of BiOCl (200) and (102), respectively. Therefore, these BiOCl nanosheets can be considered as enclosed by dominat {010} facets. In theTEM and HR-TEM images of 1% Bi_2_WO_6_–BiOCl (Fig. 3g,h), it can be clearly observed that some Bi_2_WO_6_ nanoparticles were anchored on the surface of BiOCl nanosheets. This result further demonstrated that Bi_2_WO_6_–BiOCl heterojunctions was formed in the composite. The formation of intimate interface contact is significant for promoting the charge separation to achieve high photocatalytic activity.

**Figure 4:**
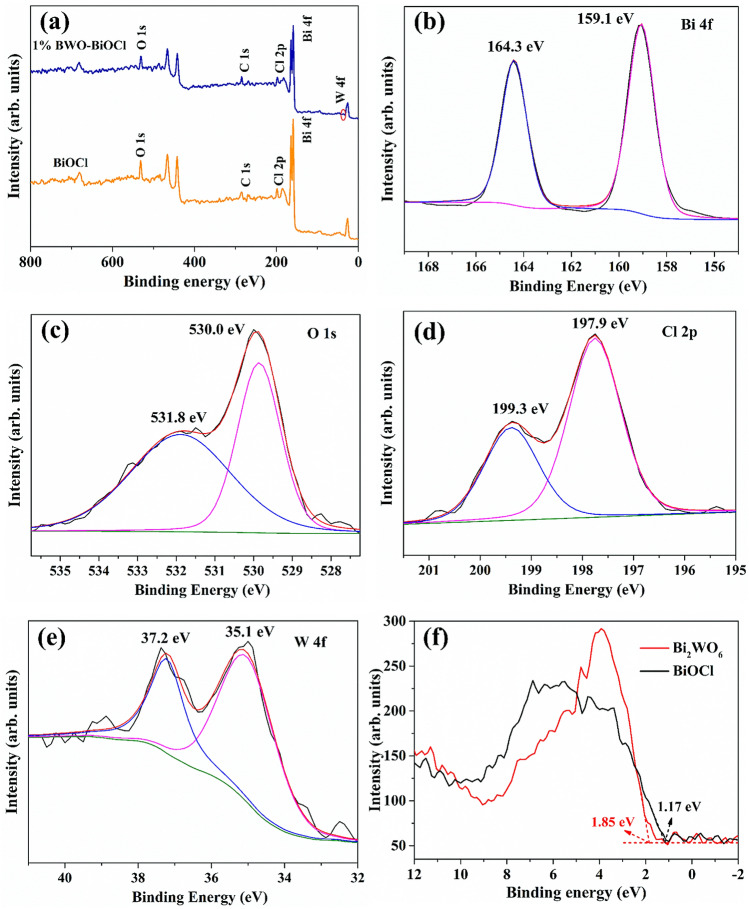
(**a**) XPS spectra of BiOCl and 1% Bi_2_WO_6_–BiOCl nanosheet, and high revoluion XPS of (**b**) Bi 4f., (**c**) O 1 s, (**d**) Cl 2p, (**e**) W 4f. for 1% Bi_2_WO_6_–BiOCl, and (**f**) XPS valence band spectrums of BiOCl and 1% Bi_2_WO_6_–BiOCl.

The surface element compositions, metal oxidation states, and valence state of 1% Bi_2_WO_6_–BiOCl as well as the pure BiOCl were further characterized by XPS. All of the data were calibrated by C as reference (284.6 eV). As shown in Fig. 4a, the XPS spectra of pure BiOCl was composed of Bi, O, Cl peaks, and the 1% Bi_2_WO_6_–BiOCl samples was comprised of Bi, O, Cl and W. The high-resolution XPS spectra of 1% Bi_2_WO_6_–BiOCl are further displayed in Fig. 4b–e. The Bi 4f. XPS spectra of 1% Bi_2_WO_6_–BiOCl was deconvoluted in two peaks with binding energies (BE) of 159.1 and 164.3 eV, which were assigned to the Bi^3+^ 4f_7/2_ and Bi^3+^ 4f_5/2_ signals^37,38^. In Fig. 4c, O^2−^ 1 s peak located at 530.0 and 531.8 eV can be attributed to the surface lattice oxygen of 1% Bi_2_WO_6_–BiOCl and binding hydroxyls of the water attached onto the surface^39,40^. In addition, the Cl 2p peaks associated with the bingding energy at 199.3 eV and 197.9 eV were all indexed to Cl^−^ 2p_3/2_^41^. While the peaks in Fig. 4e at 35.1 and 37.2 eV were attributed to the surface W^6+^ 4f_7/2_ and W^6+^ 4f_5/2_, indicating the existence of W^6+^ oxidation state^42^.

**Figure 5:**
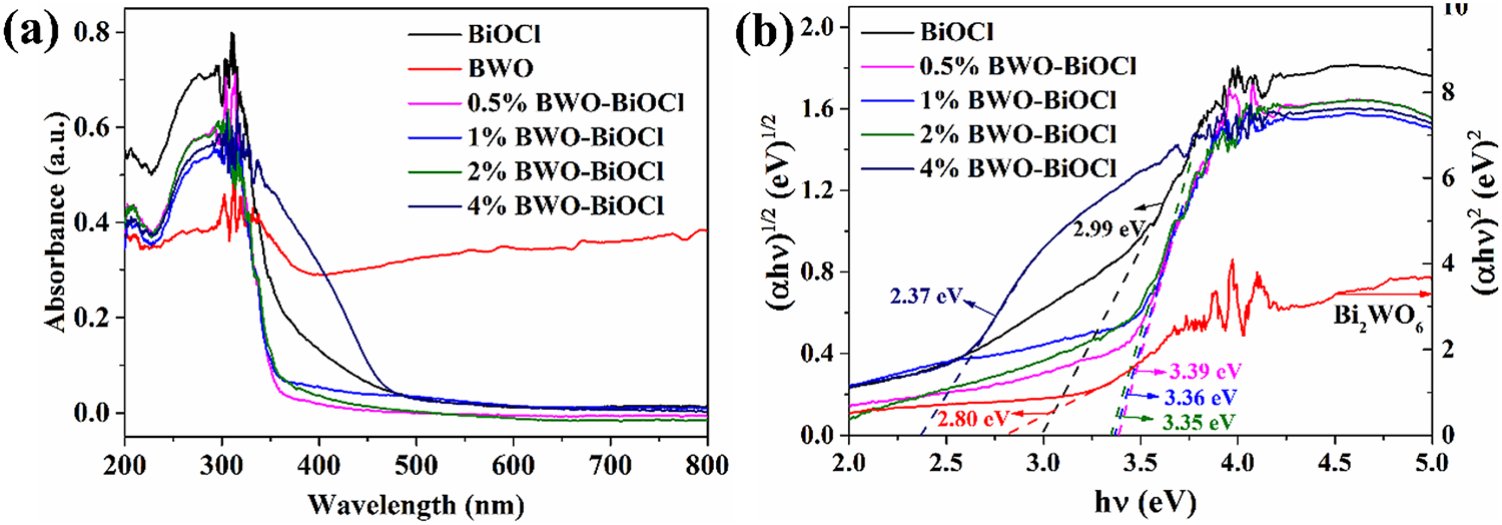
(**a**) UV–Vis diffuse reflectance spectra and (**b**) (α*h*ν)^2/n^ versus *h*ν plots attached band gap values of BiOCl, Bi_2_WO_6_, 0.5%, 1%, 2%, and 4% Bi_2_WO_6_–BiOCl.

The specific surface area and porosity of BiOCl and Bi_2_WO_6_–BiOCl were measured by the N_2_ adsorption–desorption method. Compared with BiOCl (26.6 m^2^ g^−1^), surface area of 1% Bi_2_WO_6_–BiOCl composite was relatively lower with a value of 14.8 m^2^ g^−1^, probably due to the deposition of Bi_2_WO_6_. The UV–Vis diffuse reflectance spectra (UV–Vis DRS) were conducted to determine the band gap energies of as-prepared samples. The pure BiOCl was a typical wide-band-gap semiconductor with an absorbtion edge about 415 nm (Fig. 5). A similar blue-shift of the absorption edge of 0.5%, 1%, 2% Bi_2_WO_6_–BiOCl nanosheets could be observed when comparing with BiOCl, and the absorbtion edge of 4% Bi_2_WO_6_–BiOCl showed a obvious red-shift. Based on UV–Vis DRS, the band gap of as-prepared samples can be estimated according to the Kubelka–Munk equation^43^:

1 $$\left( {\alpha h\nu } \right)^{{2}} = {\text{A}}\left( {h\nu - E_{g} } \right)^{{\text{n}}}$$

where α, *h*, ν, A, and *E*_*g*_ were absorption coefficient, Planck’s constant, light frequency, a constant, and band gap energy, respectively. The value of n was 4 for BiOCl due to its indirect transition^44^, and n = 1 for Bi_2_WO_6_ since it was a direct-gap semiconductor^45^. By extrapolating the linear portion of the (α*h*ν)^2/n^ versus *h*ν curves to the energy axis at (α*h*ν)^2/n^ = 0, the corresponding *E*_*g*_ value were calculated to be 2.99, 2.80, 3.39, 3.36, 3.35 and 2.37 eV for BiOCl, Bi_2_WO_6_, 0.5%, 1%, 2%, and 4% Bi_2_WO_6_–BiOCl, respectively.

Based on the valence band X-ray photoelectron spectra (Fig. 4f), the valance band maximum (VBM) of the BiOCl and Bi_2_WO_6_ were estimated to be 1.17 and 1.85 eV *vs* NHE, respectively. According to the relation of E_CB_ = E_VB_ − E_g_, the conduction band minimum (CBM) of BiOCl and Bi_2_WO_6_ were estimated to be − 1.82 and − 0.95 eV *vs* NHE, respectively. These data indicated that the CBM and VBM of BiOCl and Bi_2_WO_6_ were at suitable positions to construct a heterojunction structure.

### Photocatalytic activity and stability

**Figure 6:**
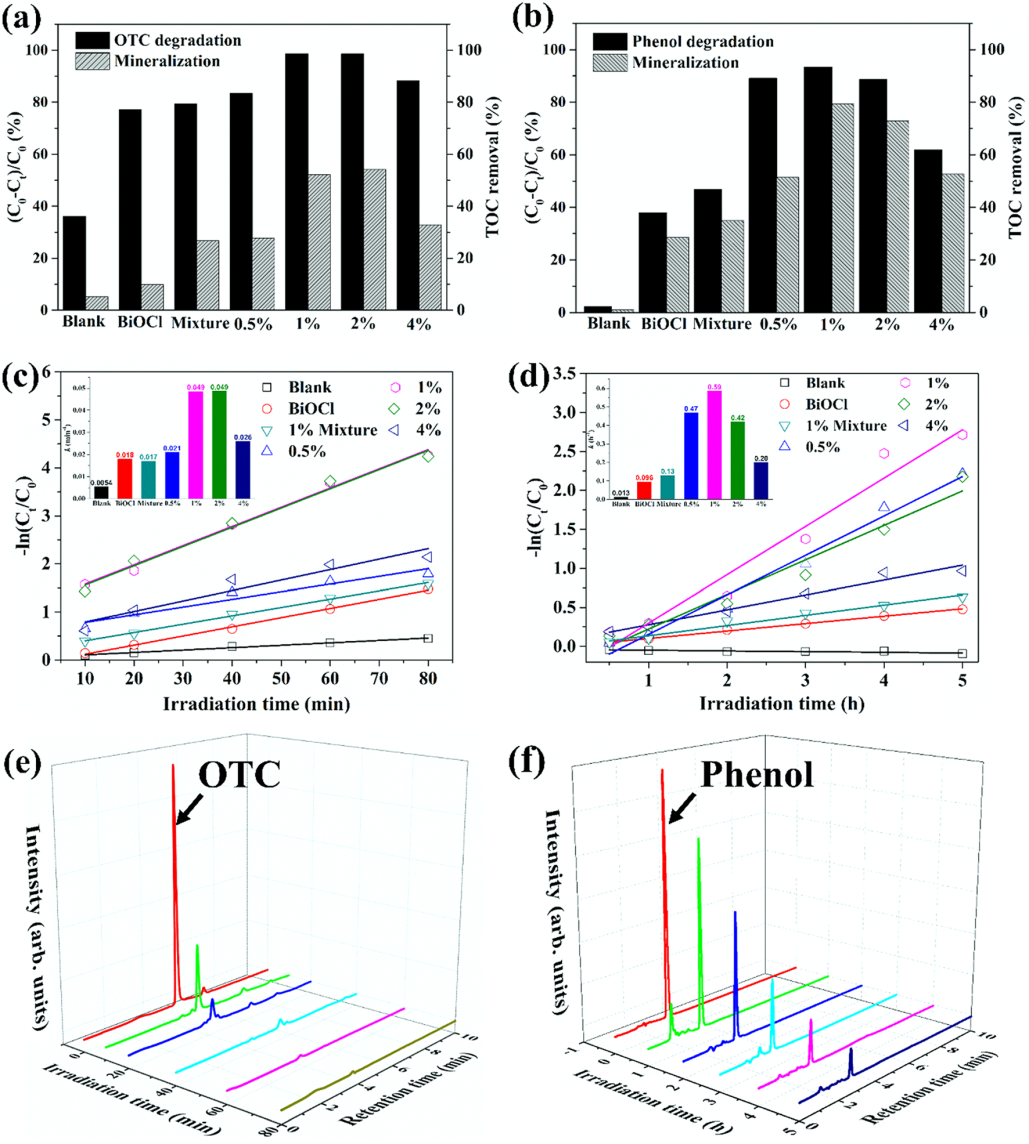
Photocatalytic degradation and mineralization rates of (**a**) OTC and (**b**) phenol, degradation kinetics of (**c**) OTC and (**d**) phenol over BiOCl, 1% mixture, 0.5%, 1%, 2%, and 4% Bi_2_WO_6_–BiOCl composites, time-dependent HPLC chromatograms of (**e**) OTC and (**f**) phenol degradation over 1% Bi_2_WO_6_–BiOCl.

The photocatalytic activities of the as-prepared samples were evaluated by degrading oxytetracycline (OTC) and phenol (20 ppm for each compound) under simulated sunlight irradiation. The 1% Bi_2_WO_6_–BiOCl exhibited the highest photocatalytic acticity with 98.6% removal of OTC after 80 min, while removal rates of 77.2%, 83.4%, 98.5%, and 88.3% can be observed for BiOCl, 0.5%, 2%, and 4% Bi_2_WO_6_–BiOCl composites, respectively (Fig. 6a). More excellent performace can be observed for the degradation of phenol (Fig. 6b,d). No phenol degradation could be found in blank treatment under the simulated sunlight irradiation, indicating the stability of phenol. The 1% Bi_2_WO_6_–BiOCl exhibited the highest photocatalytic activity with 93.4% removal of phenol after 5 h. In addition, Bi_2_WO_6_ and BiOCl were simply mechanical mixed with a ratio of 1% (denoted as 1% mixture) for comparsion, and the phtocatalytic activity toward OTC declined to 79.4%. This further implied that the heterojunction was formed between the interfaces of Bi_2_WO_6_ and BiOCl, thus leading to the favorable photocatalytic performance.

In addition, the liner relationship of − ln(C/C_0_) *vs* irradiation time (t) (− ln(C/C_0_) = *k*t) was investigated to simulate the degradation knietics. In this equation, C_t_, C_0_, and *k* were the OTC concentration after a certain reaction time (t), initial OTC concentration, and apparent rate constant (min^−1^), respectively. The photocatalysis of OTC were fitted well with pseudo first order reaction kinetics model. As shown in Fig. 6c, The rate constants (*k*) was 0.018, 0.017, 0.021, 0.049, 0.049, and 0.026 min^−1^ for BiOCl, 1% mixture, 0.5%, 1%, 2% and 4% Bi_2_WO_6_–BiOCl. To further explore the intrinstic photoreactivity, apparent reaction rate constant (*k*) was normalized to the surface area, referred to *k*_*s*_. The OTC degradation normalized reaction rate constant (*k*_*s*_) of 1% Bi_2_WO_6_–BiOCl ( 33.1 × 10^–4^ min^−1^ g m^−2^) was 3.9 folds greater than that of BiOCl (6.8 × 10^–4^ min^−1^ g m^−2^). Similarly, the *k*_*s*_ for phenol degradation of 1% Bi_2_WO_6_–BiOCl (39.9 × 10^–3^ h^−1^ g m^−2^) was also 10.1 folds higher than that of BiOCl (3.6 × 10^–3^ h^−1^ g m^−2^). The excellent photocatalytic performance of 1% Bi_2_WO_6_–BiOCl under simulated sunlight irradiation can be also visualized through the time-dependent HPLC of OTC and phenol (Fig. 6e,f).

The mineralization rate, which was evaluated as the removal of total organic carbon (TOC), is crucial for evaluating photocatalyst performance. Similar with the degradation efficiency, the highest mineralization rate was measured for 1% Bi_2_WO_6_–BiOCl with 52.2% of TOC romoval after 80 min under sunlight irradiation, which was 5.3-fold relative to that of pure BiOCl and 1.9 times that of 1% mixture (Fig. 6a). Similarly, the TOC values of phenol solution dramatically decreased from 18.63 to 1.98 mg L^−1^ after 5 h of sunlight irradiation, corresponding to 79.4% TOC removal, which was 2.8 times of that of BiOCl and 2.3 times that of 1% mixture (Fig. 6b). In addition, the photocatalytic degradation performance of phenol in related works is summarized for comparison (Table 1). Based on the parameters of degradation rate, it is obvious that 1% Bi_2_WO_6_–BiOCl sample in the current study exhibited favorable photocatalytic degradation and mineralization performance.

**Table 1:** Summary of related photocatalyst systems for phenol degradation.

Photocatalyst	Light source	Phenol (ppm)	*K *(h^−1^)	Degradation (%)	Mineralization (%)	References
Bi_2_WO_6_/BiOCl 1 g/L	500 W Xe	20	0.59	93% (5 h)	79.4%	This work
C_60_/BiOCl 1 g/L	500 W Xe	20	0.26	97% (12 h)	–	^46^
BiOCl/Bi_12_O_17_Cl_2_ 0.6 g/L	500 W Xe	10	–	46% (4 h)	–	^47^
PDI/Bi_2_WO_6_ 0.5 g/L	500 W Xe > 420 nm	5	0.36	67% (2 h)	–	^48^
BiOBr_0.9_I_0.1_/BiOI 0.8 g/L	500 W Xe > 420 nm	10	0.088	50% (8 h)		^49^
TiO_2_/BiOCl 1 g/L	300 W Xe > 450 nm	50	–	55% (6 h)	50%	^50^
BiOCl/Bi_2_MoO_6_ 1 g/L	300 W Xe > 420 nm	10	–	40% (4 h)	–	^51^
Bi_2_WO_6_/RGO 0.5 g/L	Natural sunlight	10	–	65% (8 h)	39.7%	^52^

**Figure 7:**
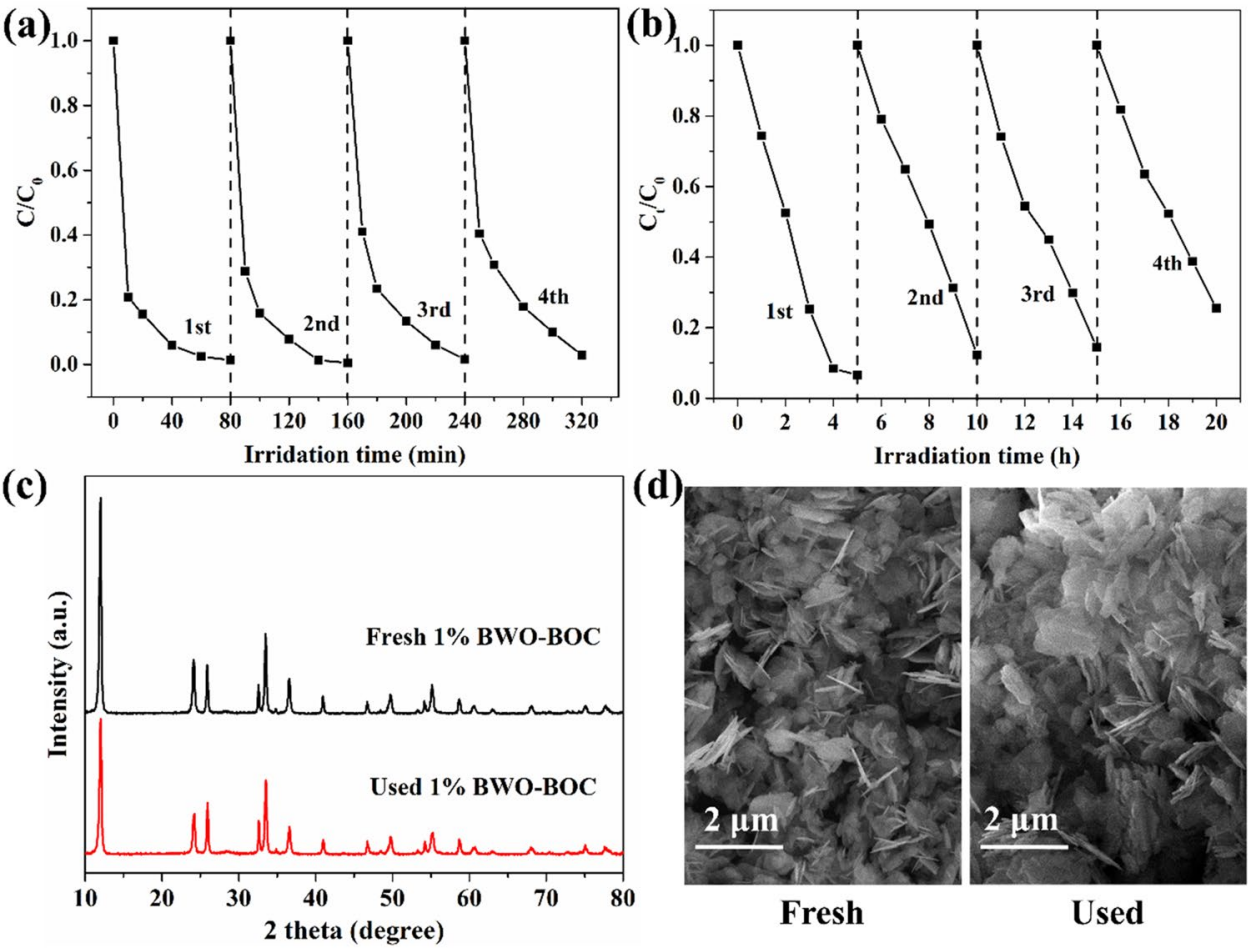
Recycling tests of 1% Bi_2_WO_6_–BiOCl for (**a**) OTC (C_0_ = 20 ppm), (**b**) phenol (C_0_ = 20 ppm) photocatalytic degradation under simulated sunlight irradiation, (**c**) XRD patterns and (**d**) SEM images of the fresh and used (20 h) 1% Bi_2_WO_6_–BiOCl for phenol degradation.

The stability of the 1% Bi_2_WO_6_–BiOCl nanosheets was investigated by cyclings of photodegradation tests. No apparent deactivation could be observed for OTC and phenol degradation after four test cycles (Fig. 7a,b). Morever, there was no significant changes between the fresh and used 1% Bi_2_WO_6_–BiOCl samples through XRD pattern diffraction peaks (Fig. 7c) and SEM images (Fig. 7d), suggesting its favorable stability for the photocatalytic decomposition of environmental contaminants.

### Mechanism of photocatalytic activity enhancement

**Figure 8:**
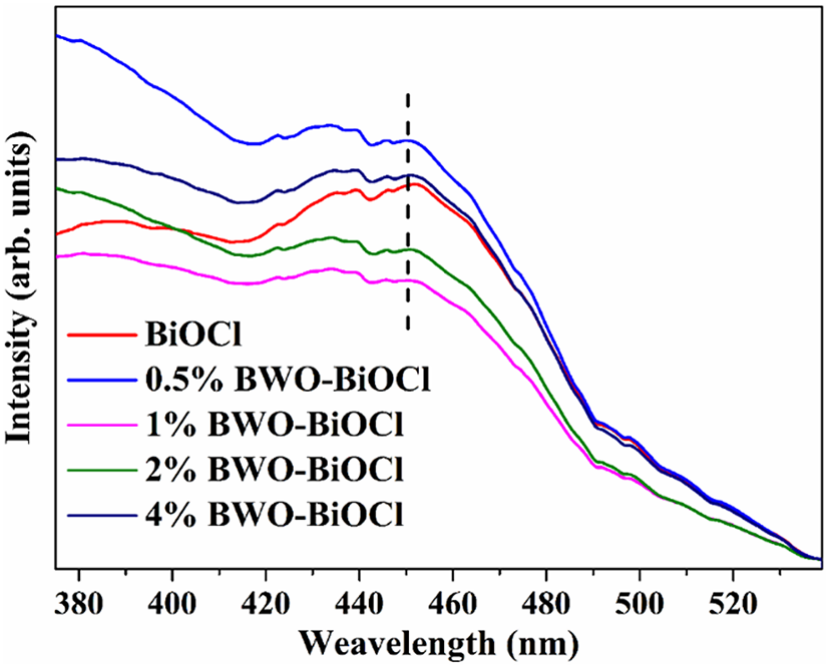
Photoluminescence (PL) spectra of BiOCl, 0.5%, 1%, 2%, and 4% Bi_2_WO_6_–BiOCl.

The recombination of photogenerated electron–hole pairs is the primary cause for the emission of photoluminescence (PL). A higher PL intensity indicates a higher recombination rate of photoexcited electron–hole pairs^53^. As shown in Fig. 8, the pure BiOCl exhibited the strongest photoluminescence intensity, while the PL intensity of Bi_2_WO_6_–BiOCl samples decreased with the introduction of Bi_2_WO_6_. The weakest PL intensity was observed for 1% Bi_2_WO_6_–BiOCl sample. It therefore can be concluded that the Bi_2_WO_6_–BiOCl heterojunctions could efficiently inhibit the recombination of photoexcited charge carriers.

**Figure 9:**
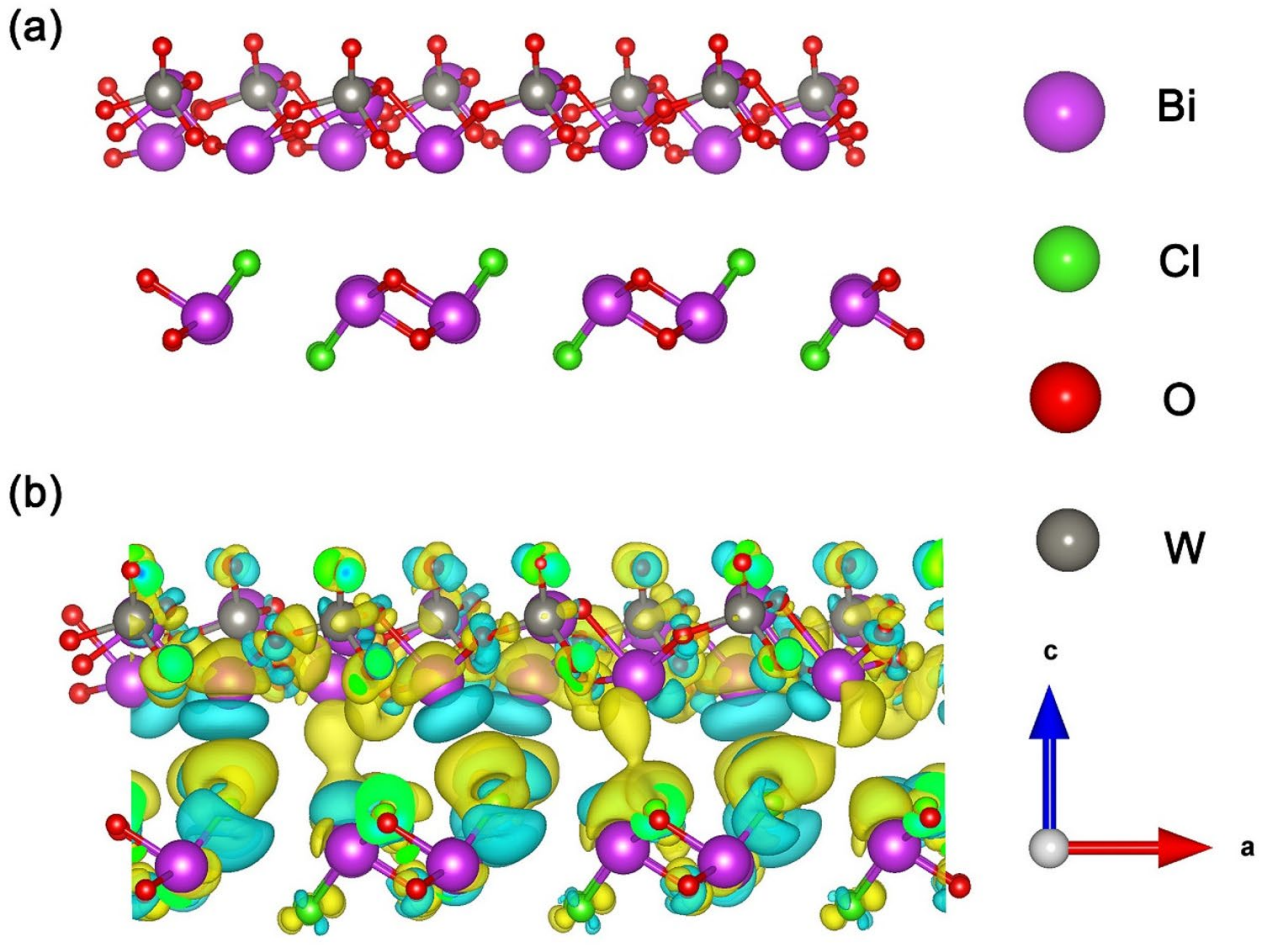
(**a**) Optimized crystal models, the Bi, Cl, O and W atoms are represented as purple, green, red and sliver, respectively. (**b**) Charge density difference map for the interfaces between Bi_2_WO_6_ (020) and BiOCl (010), the yellow region repersents charge accumulation and the cyan region indicates charge depletion.

On the basis of the HR-TEM results, a theoretical heterojunction model through contacting Bi_2_WO_6_ (020) plane and BiOCl (010) plane were constructed to investigate the charge transfer mechanism between Bi_2_WO_6_ and BiOCl interface. A 3 × 4 Bi_2_WO_6_ (020) surface slab and a 7 × 3 BiOCl (010) surface slab were matched to build the optimized Bi_2_WO_6_ (020)/BiOCl (010) interface model with an energy minimization (Fig. 9a). The Bi_2_WO_6_ consisting of [Bi_2_O_2_] layers sandwiched between two slabs of [WO_4_], equally [Bi_2_O_2_] and [Cl] layers intersected form BiOCl in the optimized crystal models, which were in good agreement with previous reports^7^.

To visualize the electron distribution the electron distributions, the electron difference density (EDD) maps and Bader charge analysis for Bi_2_WO_6_ (020)/BiOCl (010) interface were performed. The EDD mappings showed that there was an interlacing behavior between the electron rich and deficient areas. Figure 9b showed that electrons were deplected from Bi and O atoms of Bi_2_WO_6_ (as shown in cyan region), while they were accumulated in the BiOCl (as shown in yellow region), which indicated that electrons from Bi_2_WO_6_ could flow into BiOCl.

Combining with Bader charge analysis (Table 2), it can be summarized that the electron density of Bi_2_WO_6_ (020) plane was more negative than that of BiOCl (010) plane in the Bi_2_WO_6_ (020)/BiOCl (010) interface. This electron distribution resulted in a heterojunction interface electric field pointed from Bi_2_WO_6_ (020) to BiOCl (010) along the (010) direction. The built-in electric field at the interface served as a driving force to rapidly separate the photo-generated electrons and enhance photocatalytic activity.

**Table 2:** Bader charge population on the Bi, O, Cl, and W for Bi_2_WO_6_ (020)/BiOCl (010) heterojunctions.

Species	Bader charge analysis			
	Bi	O	Cl	W
BiOCl (010)	1.75	− 1.12	− 0.63	–
Bi_2_WO_6_ (020)	1.80	− 1.04	–	2.64

**Figure 10:**
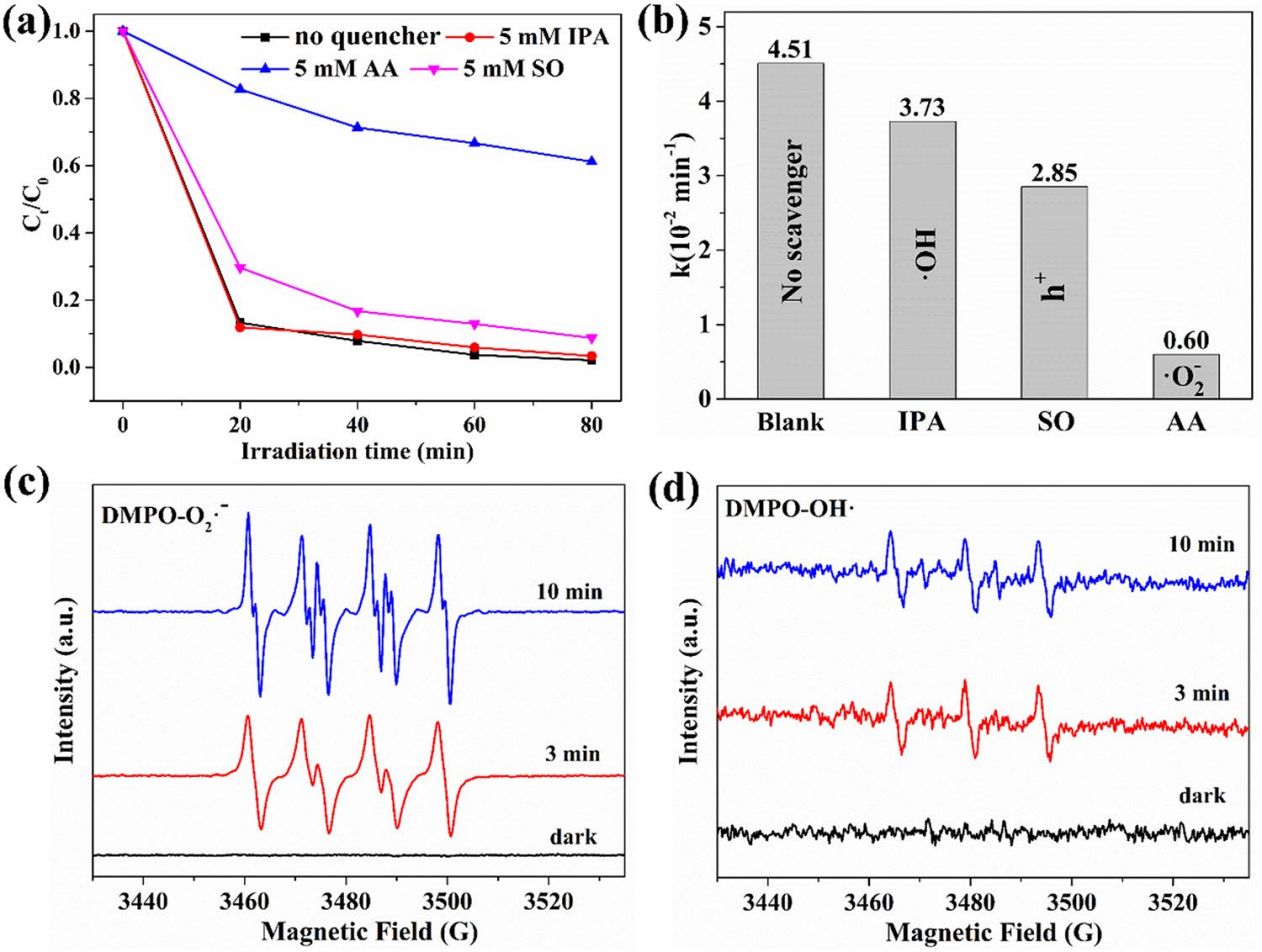
(**a**) Photocatalytic degradation of OTC over 1% Bi_2_WO_6_–BiOCl in the presence of scavengers under simulated sunlight irradiation, (**b**) Psedo first-order kinetic fitting and the determined apparent rate constants (*k*) with different quenchers, ESR spectra of (**c**) DMPO-^·^O_2_^−^ , and (**d**) DMPO-^·^OH over 1% Bi_2_WO_6_–BiOCl samples.

The reactive species trapping experiments were conducted to further explore the possible photocatalytic mechanisms over the 1% Bi_2_WO_6_–BiOCl sample. Ascorbic acid (AA, 5 mM), isopropanol (IPA, 5 mM), and sodiun oxalate (SO, 5 mM) were used as scavengers for superoxide radical (^·^O_2_^−^), hydroxyl radical (^·^OH), and holes (h^+^)^54^, respectively. The OTC conversion slightly decreased with the addition of IPA and SO (Fig. 10a). In contrast, OTC conversion significantly dropped from 98.9% to 38.8% within 80 min of sunlight illumination when AA were added. The photocatalytic degradation pseudo-first order kinetics constant of OTC decreased from 0.0415 to 0.0373, 0.0285 and 0.006 min^−1^ with adding of IPA, SO and AA (Fig. 10b). This demonstrated that the photocatalytic process was mainly governed by the ^·^O_2_^−^, and the role of h^+^ cannot be ignored in this system.

ESR spin trap technique was employed to study the main reactive oxygen species generated by 1% Bi_2_WO_6_–BiOCl. The 5,5-dimethyl-1-pyrroline N-oxide (DMPO) was used to capture the ^·^O_2_^−^ and ^·^OH. After irradiation for 3 min and 10 min, the special spectrum with an intensity ratio of 1:1:1:1 quartet signal was obviously observed in 1% Bi_2_WO_6_–BiOCl (Fig. 10c), which was ascribed to the characteristic spectrum of DMPO-^·^O_2_^−^ adduct^55^. In addition, the ESR signal increased with irradiation time prolonging from 3 to 10 min. At the same time weak DMPO-^·^OH adduct ESR signals with the relative intensities of 1:2:2:1 were detected over the 1% Bi_2_WO_6_–BiOCl under the irradiation of sunlight (Fig. 10d). Considering the results of reactive species trapping experiments and ESR characterization, it can be inferred that ^·^O_2_^−^ was the main active species in the photocatalytic process over the 1% Bi_2_WO_6_–BiOCl.

**Figure 11:**
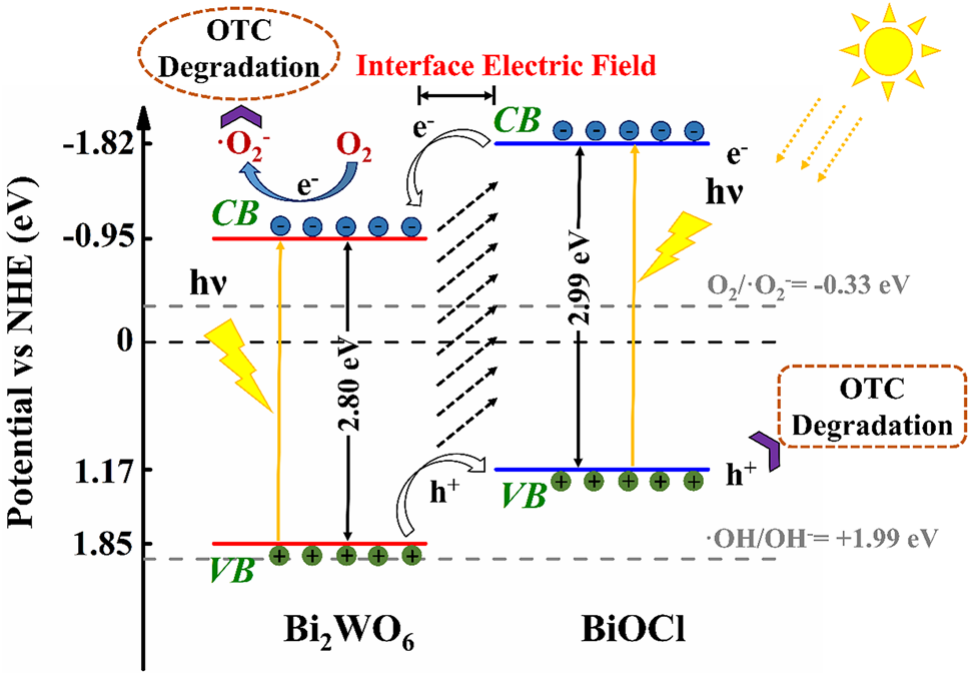
Schematic illustration of band structure diagram and photinduced carriers transfer of 1% Bi_2_WO_6_–BiOCl composites under sunlight irradiation.

The schematic diagrams for CBM and VBM electrochemical potentials of BiOCl and Bi_2_WO_6_ as well as the possible charge separation process of 1% Bi_2_WO_6_–BiOCl are shown in Fig. 11. The CBM potentials (− 1.82 and − 0.95 eV *vs.* NHE) of BiOCl and Bi_2_WO_6_ were more negative than the standard redox potential of O_2_/^·^O_2_^−^ (− 0.33 eV *vs* NHE, pH 7)^56^. The more negative potential than ^·^O_2_^−^ radical allowed the yield of ^·^O_2_^−^ via reduction of adsorbed O_2_ by photogenerated e^−^. The VBM potentials for Bi_2_WO_6_ and BiOCl (1.85 and 1.17 eV *vs.* NHE) were more negative than the standard redox potential edge of ^·^OH/OH^−^ (+ 1.99 eV *vs* NHE, pH 7) and ^·^OH/H_2_O (+ 2.27 eV eV *vs* NHE, pH 7), indicating that the photogenerated holes cannot directly oxide H_2_O molecules to ^·^OH radicals^57^.

Based on the above characterizations and DFT calculation results, a possible photocatalytic mechanism for 1% Bi_2_WO_6_–BiOCl is depicated in Fig. 11. The BiOCl and Bi_2_WO_6_ were exicited simultaneously under simulated sunlight irradiation, the electrons in the VBM were excited into the CBM, and the same amount of holes (h^+^) were remained in the VBM. Since the CBM potential of BiOCl (−1.82 eV) was more negative than that of Bi_2_WO_6_ (− 0.95 eV), the photinduced electrons on the interface of BiOCl can migrate to the CBM of Bi_2_WO_6_ by the heterojunction interface in the composite system. Similarly , photogenerated holes on the Bi_2_WO_6_ surface can migrate to VBM of BiOCl. The electrons in the Bi_2_WO_6_ could be captured by the adsorbed O_2_ to yield ^·^O_2_^−^ radicals. While the photogenerated holes concentrated on the surface of BiOCl, achieving the efficient separation of the photoinduced electrons and holes on the heterojunction. Moreover the existence of internal electric field could further promote the efficient transfer of photogenerated carriers, thus leading to enhanced photocatalytic activity. The whole process can be described by the following equations:

2 $${\text{1}}\% {\text{Bi}}_{{\text{2}}} {\text{WO}}_{{\text{6}}} - {\text{BiOCl}}\mathop \to \limits^{{{\text{h}}\nu }} {\text{Bi}}_{{\text{2}}} {\text{WO}}_{{\text{6}}} {\text{ (e}}^{ - } {\text{,h}}^{{\text{ + }}} {\text{)/BiOCl (e}}^{ - } {\text{,h}}^{{\text{ + }}} {\text{)}}$$

3 $${\text{Bi}}_{2} {\text{WO}}_{6} {\text{ }}({\text{e}}^{ - } ,{\text{h}}^{ + } )/{\text{BiOCl }}({\text{e}}^{ - } ,{\text{h}}^{ + } )\mathop \to \limits^{{{\text{hetero}} - }} {\text{Bi}}_{2} {\text{WO}}_{6} \left( {{\text{e}}^{ - } } \right) + {\text{BiOCl }}({\text{h}}^{ + } )$$

4 $${\text{Bi}}_{2} {\text{WO}}_{6} \left( {{\text{e}}^{ - } } \right){\text{ + O}}_{2} \to {\text{Bi}}_{2} {\text{WO}}_{6} {\text{ + }}^{ \cdot } {\text{O}}_{2}^{ - }$$

5 $$^{ \cdot } O_{2}^{ - } {\text{ + OTC}} \to {\text{products}}$$

6 $${\text{BiOCl (h}}^{ + } ){\text{ + OTC}} \to {\text{products}}$$

## Conclusions

The BiOCl nanosheets and Bi_2_WO_6_–BiOCl composites were successfully synthesized by the facile hydrothermal and solvothermal process. The photocatalytic activities and mineralization rates of 1% Bi_2_WO_6_–BiOCl for OTC and phenol were superior to individual BiOCl under simulated sunlight irradiation. The OTC and phenol degradation rates was almost 2.7 and 6.1 times as that of BiOCl, and the mineralization rate of OTC and phenol was 5.3 and 2.8 folds relative to that of BiOCl. The favorable photocatalytic performance was attributed to the synergistic effect of proper bandgap matching, and efficient separation of photogenerated charge carriers as a result of heterojuntion interface effect between BiOCl and Bi_2_WO_6_, which was verified by the experimental characterizations and DFT calculations. Further experiments demonstrated that the photocatalysis degradation of OTC was due to the oxidation of superoxide radical. Cyclic sunlight irradiation experiments demonstrated the reusability and stability of 1% Bi_2_WO_6_–BiOCl. Therefore, it can be concluded that the Bi_2_WO_6_–BiOCl heterojunction photocatalyst is a promising candidate for photocatalytic decomposition of organic contaminants.
